# Barriers to and Facilitators of Physical Activity: A Qualitative Study from the Perspective of Individuals Living with Sight Loss in Cambridgeshire

**DOI:** 10.3390/vision7040070

**Published:** 2023-11-02

**Authors:** Olivia Hillan, Lee Smith, Simon Bishop, Peter M. Allen

**Affiliations:** 1 Centre for Health, Performance and Wellbeing, Anglia Ruskin University, Cambridge CB1 1PT, UK; oh13@aru.ac.uk (O.H.); lee.smith@aru.ac.uk (L.S.); 2 School of Medicine, Anglia Ruskin University, Chelmsford CM1 1SQ, UK; simon.bishop@aru.ac.uk; 3 Vision and Hearing Sciences Research Centre, Anglia Ruskin University, Cambridge CB1 1PT, UK

**Keywords:** physical activity, visual impairment, qualitative methods

## Abstract

Physical inactivity is a prevalent concern amongst adults living with sight loss. It is essential to understand why these individuals are inactive and how we can increase physical activity levels among them. Therefore, this study aims to explore the barriers and facilitators to physical activity for individuals living with sight loss. Seven individuals with self-reported sight loss living in Cambridgeshire were recruited for three focus groups. Focus group data were analysed using thematic analysis to identify key themes. Seven themes which represented a barrier, or a facilitator were identified: transport, accessing information, one size fits all, negative previous experience, visually impaired sport, women in disability sport, and taster days. To increase physical activity levels amongst those living with sight loss, interventions need to be focused on the organisational level. This includes producing more accessible environments that can be produced by providing training for sport and physical activity professionals and by ensuring the physical environment is inclusive for those living with sight loss.

## 1. Introduction

Globally, there are an estimated 2.2 billion people living with a visual impairment (VI) [1]. In the UK, this equates to approximately 2 million people living with sight loss, of whom 22,900 are living in Cambridgeshire [2]. This includes those who are registered as blind or partially sighted, as well as those with less severe sight loss. This number is expected to rise with predictions that approximately 4 million people in the UK will be living with sight loss by 2050 [3].

VI can impact on all aspects of a person’s life and has been consistently linked to poorer physical health. Court et al. [4] reported that 27 out of 29 physical health conditions including hypertension, coronary artery disease, and diabetes were more prevalent amongst those with a VI. Additionally, those living with a VI were twice as likely to have five or more physical or mental health comorbidities compared to sighted participants [4]. Importantly, when compared to individuals without VI, risk of mortality is 29% greater for people with mild VI and 89% greater for those with severe VI [5].

Sight loss is also linked with poorer mental health with 40% of people with sight loss feeling “moderately” or “completely” cut off from people and things around them [6]. Additionally, one in four adults with sight loss report anxiety or depression and sight loss is now a recognised risk factor for depression [4,7,8].

One strategy to improve physical and mental health for people living with sight loss is to increase physical activity (PA) levels. PA is defined as “any movement produced by skeletal muscles that requires energy expenditure—including activities undertaken while working, playing, carrying out household chores, travelling, and engaging in recreational pursuits” [9]. Currently, the World Health Organisation recommends at least 150–300 min of moderate intensity PA or 77–150 min of vigorous PA per week [10]. They also recommend muscle strengthening activities that involve all major muscle groups should be completed at least twice a week. These recommendations are the same for adults with disabilities [10].

Regular PA is consistently linked with the effective management of several chronic conditions [11,12] as well as improving the symptoms of anxiety and depression [13,14]. Research has also reported regular PA can improve functional independence, quality of life, and prevent the risk of falls amongst adults with a VI [15].

Despite the known benefits of PA, physical inactivity remains a global concern. Levels of inactivity are more prevalent amongst those living with sight loss. Smith et al. [16] found that, compared to those who reported having excellent vision, those who rated their eyesight “fair–poor” were more than twice as likely to self-report being physically inactive. Therefore, it is essential to increase PA levels amongst those living with sight loss to help reduce the current health inequalities. A recent study from the Royal National Institute of Blind People determined that one in three blind and partially sighted people have sports or fitness activities they would like to try but have not been able to [17]. Understanding the barriers to accessing PA will help facilitate Jones et al.’s [18] recent call for action to increase awareness and promote collaboration between people with VIs and service providers to facilitate safer and more accessible fitness environments. Limited research has explored the barriers to PA participation amongst those living with sight loss and all current research has focused on children or adults aged over 65 and fails to represent the working-age population [19,20,21,22]. Therefore, this study aimed to explore the barriers and facilitators to PA participation among people with sight loss, including the working-age population.

## 2. Materials and Methods

Following institutional ethical approval participants were recruited using a purposive sampling technique, followed by snowball sampling, between January 2023–June 2023. The study was advertised using local sight loss charities social media pages and mailing lists which allowed interested participants to contact the researcher directly. Interested participants were then sent an information sheet detailing what their participation would involve. Participants who chose to participate were then sent a link to an online participant demographic survey using JiSC surveys (results presented in Table 1). This was used due its accessibility for those who use screen readers. Participants were included if they met the following criteria: (1) aged 18 years or older; (2) have sight loss which cannot be corrected by spectacles or contact lenses; (3) live in Cambridgeshire, UK. Recruitment continued until the researchers had identified the main barriers and facilitators to physical activity, no new themes were identified, and it was deemed that the data were rich and had conceptual depth [23]. It was deemed that each theme provided sufficient conceptual depth based on Nelson’s [24] criteria for conceptual depth criteria and all themes provided a meaningful insight into the research question. Three focus groups were conducted via video call and lasted approximately 60 min. The first focus group consisted of three participants, with the following two consisting of two participants. Three focus groups were deemed appropriate in line with the study’s qualitative design and because small samples can still substantially capture and reflect people’s experiences [25]. The focus groups were held online in a deliberate attempt to make them more accessible and help reduce any travel barriers for participants. The researcher had no previous relationship with the participants and all participants provided digital consent using electronic consent forms. Consent was confirmed verbally before the start of each focus group. This was approved by our ethics committee. The focus group began by discussing the purpose of the focus group, reminders about anonymity, and allowing participants to ask any questions. Participants were then asked questions specifically aimed at the barriers and facilitators to PA such as “what key things helped you to get involved with physical activity?” and “what do you think are the barriers to being physically active in your local area?”. Follow-up questions and probes were used to ensure a rich understanding of the topic [26]. The full focus group guide can be found in the Supplementary Materials. Focus groups were audio-recorded and transcribed verbatim. Data were analysed using inductive thematic analysis [27]. Firstly, focus groups were transcribed and read repeatedly. After this familiarisation phase, phrases were coded, and initial concepts were noted. The coded data were then collated, and potential themes were identified. These themes were then reviewed and refined to ensure they accurately represented the data in relation to the research question. The final themes were discussed with all authors to ensure they could be justified [28].

## 3. Results

The final sample consisted of seven visually impaired adults currently aged between 18 to 74 years of age (Mean = 36, SD = 18 years) who self-reported as partially sighted or severely sight impaired (blind). All participants classed themselves as physically active.

Thematic analysis generated seven themes that helped to explain the challenges or facilitators to participating in PA. Often themes represented a barrier and a facilitator. The final themes included: transport (cost and reliability), accessing information, one size fits all, negative previous experience, visually impaired sport, women in disability sport, and taster days. Each theme will be discussed in turn.

### 3.1. Theme—Transport: Cost and Reliability

Transport was the most frequently discussed barrier to regular PA participation. Whilst having a concessionary travel pass was often recognised as an important facilitator, public transport is infrequent and unreliable particularly for those living in rural areas. This meant that despite an enthusiasm to be physically active often participants were unable to access their chosen activities due to the logistical challenges of attending the venue. This also contributed to reduced self-esteem and independence due to an increased reliance on those around them to transport them to training sessions, which left participants feeling like a burden on others.

*Participant 4: “but doesn’t it just suck (bad) that it means therefore…you’re not independent when you have to rely on lifts (getting dropped off by others), and you really have to haggle with the people who love you”.*

*Participant 5: “and this is why I don’t ask my parents to take me to competitions because competitions are long days… there’s no way I’m putting them through a whole day of judo just to watch me fight for 8 min”.*

In addition to the emotional consequences of unreliable public transport there is often an additional financial cost incurred. Sessions are mainly held in the city centre, which is challenging to reach for those living in the more rural areas, and training sessions regularly finish after public transport has stopped. This forces participants to use private transport (taxis) which increases the cost and *“it can turn a £5 session into a £35 session” (participant 5)* which is deemed too expensive and makes accessing PA unattainable.

Overall, participants did not express a desire to move training sessions to more rural areas but instead reinforced the need to improve local public transport systems. Notably, public transport was not a barrier exclusive to sport and was often a barrier to most aspects of participants lives, including employment and socialising with others.

### 3.2. Theme: Accessing Information about Local Opportunities

Participants shared how it can be difficult to know where to find information about local opportunities and this created the perception that is often a case *“of who you know not what you know” (participant 7)*.

*Participant 4: “if you’re trying to access sport as a solo player with a disability in any form and your family and your teachers don’t know (about the available opportunities) and nobody local and none of your club coaches know then you’re sort of stuck (being inactive)”.*

Being surrounded by individuals who are not aware of local opportunities meant often participants were but were not aware of how to access this information.

To facilitate this, one suggestion was local charities have accurate and up to date PA specific sections on their websites. Importantly it was highlighted that these websites cannot be *“territorial” (participant 7)* and only advertise their own services but instead must include all opportunities from all local providers. It was also highlighted how any advertisements must be produced in *“at least two or three different types” (participant 5)*, to ensure it is accessible for all service users. Examples included producing the same version of an advertisement but removing any photos so that it is compatible with screen readers. Additional examples included using large-print font or using talking newspapers to advertise opportunities. It was acknowledged that whilst some organisations have created activity finders which contain information about local sports clubs and training sessions, it is still a challenge to obtain information regarding opportunities in the local area, particularly if you are not aware these exist.

*Participant 7: “there are a lot of organisations out there so it can be quite difficult to know where to go (to find information about available local sporting opportunities)”.*

Participants also discussed how advertising using charities alone would be insufficient as charities often do not reach everyone who is living with a VI. Participants felt that charities tend to focus solely on younger children or older adults *“due to the nature of VI” (participant 4)*, as most people living with sight loss are aged over 65 [2]. This creates issues for the working age population who are living with sight loss as it means sessions are often delivered during working hours or are targeted specifically at these age groups.

Overall, participants felt it is difficult to access sporting opportunities as they are often not well promoted or are not advertised in an accessible format. Instead, participants are often left relying on the knowledge of others around them which means they are often unaware of potential opportunities.

### 3.3. Theme: One Size Fits All

There was a common consensus among participants that current PA opportunities operate from a *“one size fits all approach” (participant 1)* and an assumption that all individuals with a VI will want to participate in the same thing. Participants shared the belief that when organisations are offering sports that are accessible for people with a VI, often these tend to *“be the same across the board” (participant 1)*, i.e., only offering VI tennis, goalball, or yoga, and *“fails to consider what people actually want” (participant 1)*.

This approach has resulted in individuals struggling to adhere to regular PA as their desired activity is not available to them. Often the only way to participate involved increasing the cost of the activity by hiring a personal trainer or having to hire specific coaches which is deemed too expensive.

*Participant 1: “Which turns it from a free activity, which it is for everyone else, to a ridiculously expensive one for me because I happen to be totally blind”.*

For some participants accessing a session online was their preferred method of being active whereas for others this was perceived as a *“worst-case scenario” (participant 7)*, due to a personal preference of attending in-person activities. Thus, reinforcing the harmfulness in the assumption that all individuals are going to want to participate in the same activities simply because they are visually impaired.

To summarise, participants emphasised the importance of recognising individual differences and not assuming that every individual living with sight loss is going to want the same activities.

### 3.4. Theme: Negative Previous Experience

As children, participants often had negative sporting experiences which deterred them from being active as an adult. These data suggest that this stemmed from a lack of knowledge and understanding from coaches or teachers which resulted in poor coaching practice and the child stopping PA completely.

*Participant 5: “[I’ve had instructors saying], I have never had anyone not learn snowboarding after a 2-h lesson and I was 14 and I was gutted, and I never went snowboarding again.”*

School physical education lessons were often negative experiences for participants as they were left to sit on the side because their teacher did not know how to adapt the sessions. For some participants they were placed in classes with other students with disabilities, but this was not perceived to be a facilitator. Instead:

*Participant 4: “as a visually impaired person you are often lumped together with people that have learning disabilities or actual physical disabilities… and then you’re all treated the same. It’s just the stuff (e.g., physical adaptations) that you need to do for them is completely different to the stuff that you need to do for me. But then you feel like you’re being held back because you’re spending a long time being taught something that you already know how to do”.*

Having a negative experience as a child meant participants were often not physically active until they reached adulthood. However, even as an adult they frequently received negative attitudes from coaches and instructors. Participants who attended exercise classes or gym environments often had instructors who did not know how to train a person with a VI. There was a common misconception that visually impaired services users required the session to be made easier due to their VI. For example:

*Participant 2: “I did this 1-1 induction with this instructor… and we went through this whole plan and then a few days later I got a text saying unfortunately I can’t train you, but we’ve got this guy who’s pretty good with rehabilitation…. he was nice enough, but he mentioned my conditions every time we did a session, and he would say ‘do what you can’ and he literally said, ‘I don’t want to push you.’”*

Participants suggested that coaches should be given training on how to coach people with a VI. To be effective this training must not offer a checklist solution but instead recognise the importance of understanding the individual and their needs on a case-by-case basis. It is also crucial that coaches are given training that *“isn’t dependent on being able to see” (participant 5)* and instead coaches need to listen properly and make adaptions in a sensitive manner. Coaches must also be educated on the different British Blind Sport Recreational Sight Classifications which range from a B1 to a B5, with B1 being the greatest level of sight loss [29]. This is because:

*Participant 5: “what’s going to work for someone who is totally blind is completely different from someone who’s a B3 or a B4.”*

Participants also discussed times of how the coach had intended to be supportive, but this support was not perceived that way. Examples included being told *“it’s not a race, it doesn’t matter” (participant 4)* because it was taking the individual longer to learn a new skill than the rest of the class. Whilst well-meaning for the participant this was not deemed as helpful because:

*Participant 4: “It matters to me because I’m feeling inferior because everyone else seems to be able to do this thing and I can’t.”*

Overall, these data suggest that service providers must be given training on working with a person with a visual impairment to ensure they are creating a positive and inclusive environment that will encourage retention of the person in the PA sessions.

### 3.5. Theme: Visually Impaired Sport

In traditional sport or exercise classes participants found they were often the only person with a VI in the session. This resulted in participants feeling they were undeservingly requiring additional time and help from instructors. When learning a new skill:

*Participant 4: “I feel quite a lot of guilt because I have to take up so much time for the teachers and the more senior players to help me. They’re (the more senior players) then not getting the time that they need to train because then they’re still trying to get me to understand.”*

Another barrier participants often had to overcome is the negative perceptions from those around them due to visual impairments often not being visible disabilities. This extended beyond just participating in sport and participants discussed how they spend a lot of time trying to mask their disabilities, but when it comes to participating in PA this is not possible.

*Participant 4: “If you don’t look visually impaired, you have to act like an able person, because otherwise you’ll get weird comments and weird looks, so I mask an awful lot and this is why I get so anxious with sport, because I can’t mask when I do sport… when I do sport the mask comes off and then I’m really vulnerable.”*

This vulnerability then influenced the relationship with others in the club because:

*Participant 4: then I get really angry and really angsty. And then I get snappy and the coach is like, we’re just trying to help you, and if you’re gonna be like this, then we’re not gonna help you..., I don’t mean to be like this. It’s the anxiety coming out sideways.”*

For some participants the biggest facilitator to being physically active was joining VI-specific sport. This allowed participants to increase their confidence, not only in sport, but in all aspects of their life. Participants found a place where they *“felt like they belonged” (participant 6)*, that allowed them to meet *“hyper competent, capable, visually impaired people that I found my niche with” (participant 5)*. For one participant VI sport *“really normalised disability for me and destigmatised it which meant I was less self-conscious about my disability” (participant 5)*.

This sense of belonging was largely facilitated due to the shared understanding that all group members have and the importance of the support from everyone in the club.

*Participant 6: “everyone in our club is so friendly and we all get on really well and I know it’s a competitive sport, but we all support each other whether we’re on the same team or opponents”.*

*Participant 7: “I think that sense of a community around the club has built up a lot over the years as well and all the beneficial stuff that I’ve got from the sport, about learning to live with a visual impairment, I think that continues within our club in terms of the support we provide each other and it being a place where people can reach out for support as much as be a sports club.”*

### 3.6. Theme: Women in Disability Sport

Whilst VI-specific sport was a facilitator for many participants, it was identified that this was not without its barriers. Participants found that being a woman in disability sport is *“just a minority, within a minority, within a minority” (participant 5)*. Whilst these participants were maintaining regular PA, they were participating in sports that were typically dominated by men (goalball and judo). For one participant this meant they would *“describe themselves as a forever novice”* (participant 5) due to the physical differences between their male teammates. This was also associated with having to pretend they are enjoying themselves in order to continue being physically active:

*Participant 4: “I have to pretend that I love it because I want to stay with it and I want to improve and I want to get better, but really I’m sitting there thinking this is awful.”*

However, when asked if they would prefer female only sports participants were hesitant about the idea and shared: *“we have only just got to the level that we have women in the sport to even think about doing it” (participant 4)*.

Overall, VI-specific sport helped to increase PA because it provided participants with a safe space that allowed them to be physically active whilst also feeling supported and encouraged by their teammates.

### 3.7. Theme: Taster Days

Participants highlighted free sporting taster days were a good way to engage people to join a new activity. For most participants in this study a taster session or taster day was how they discovered their chosen activity. However, participants stressed the importance of authenticity when hosting these sessions, so they do not feel *“patronising” (participant 5)*. In other words, whilst enjoyment of these sessions is crucial, they must also accurately represent what the sport looks like to ensure long-term adherence. When hosting a goalball taster session, it was essential that the rules of the game are enforced, and that the environment was reflective of a typical training session:

*Participant 7: “if they see equipment like that (proper goalball goals instead of benches), if they see people wearing club kit and all these kinds of things, I think it makes it a lot more real and helps people buy into something.”*

It was unanimous amongst participants that for any new sports team, club, programme, etc., that the most important way to ensure success for its blind and partially sighted members is to have people living with sight loss involved in the organisational and decision-making processes. Having a club that is *“by the blind for the blind” (participant 5)*, with people that can offer lived experience and feedback is essential to ensure accessibility and inclusivity.

To summarise, a taster session or taster day that is led by visually impaired individuals and accurately represents the activity is a facilitator to being physically active. It allows individuals to try out an activity without feeling obliged to attend again but often, well-organised, encourages long-term attendance and adherence to PA.

## 4. Discussion

The present study explored the barriers and facilitators to PA for people living with sight loss. These included: transport, accessing information, one size fits all, negative previous experience, visually impaired sport, women in disability sport, and taster days. This study extends current literature by presenting new findings and through the inclusion of adults of a working age.

The finding that transport is one of the largest barriers to PA is in line with previous research which also reported public transport as a barrier for older adults living with sight loss [20]. This research shows that despite public transport improvements, for people with a VI, this barrier still exists and has not been addressed adequately. Thus, to ensure that PA promotion can be facilitated change is required that improves the frequency and reliability of public transport networks to ensure individuals can logistically attend their desired activity.

This study also highlights the importance of moving away from the perception that all people with a VI have the same interests and will only want to access limited sports (e.g., tennis, football, goalball, yoga). Individuals with a VI are not a homogenous group and values and interests differ significantly for all individuals [30]. This research has shown a novel finding around the idea that people with a VI try to “hide” or “mask” their VI. Masking was associated with increased feelings of anxiety and frustration. Given the high rates of anxiety and depression amongst those living with sight loss [4,7,8], it is of particular concern that some participants felt they must hide who they really are. It is essential that all PA and sporting environments are ensuring accessibility and autonomy and are creating spaces whereby no participants have to hide their true self. Regardless of the type of activity on offer it is essential that activities are well promoted, and that any advertisements are accessible. When advertising PA British Blind Sport recommend providing information in multiple formats (e.g., braille, large high-contrast print for printed information, and suitable for screen readers). Currently some sporting organisations have adapted their provision, but attendance levels are still low due to poor information provision [30].

Additionally, this research reported VI-specific sport as a facilitator for PA. This allows people with VI to find environments that help increase their feelings of self-worth and confidence. Finding an inclusive environment helps participants embrace their disability identity, which refers to self-concept as a person with a disability [31] and disability self-worth. Disability self-worth encompasses the beliefs that individuals with a disability are of equal value in society as people without physical or mental disabilities [32]. Similar findings were reported where some individuals did not consider themselves as disabled and therefore were put off by sporting opportunities that target disabled people [33]. Whereas others preferred disability sport as they found it was more accepting and understanding. Increasing disability self-worth reduces anxiety and depression [34]. Whilst the exact mechanism for why this occurs is currently unclear, the authors hypothesised that this was due to a decrease in negative affective experiences (e.g., shame, guilt) and increase in positive affective experiences (e.g., joy, pride), which supports the experiences of the participants in this study. However, disability self-worth was not measured in this study and was discussed only in relation to PA. Therefore, future research should aim to explore the influence of PA on disability self-worth, anxiety, and depression amongst those living with a VI.

The present study also reinforced the importance of challenging the negative stereotype through education and training, for those working in the PA and sport domain. A lack of knowledge among coaches, PE teachers, trainers, etc., is consistently reported amongst all age groups as a barrier to PA for those with a VI [21,35]. This lack of understanding results in harmful practices, that despite good intentions, can have negative consequences on long-term PA adherence as participants never returned to that sport. Increasing school age PA increases the probability of being active in adulthood making a positive experience essential [36].

One important finding of this study was the significance of being a visually impaired woman in sport. Participants felt they had to pretend they enjoyed playing sport with men because the alterative was not to participate. It is frequently reported that PA levels are higher amongst men with a VI compared to females [15,37,38]. Given that over 60% of people living with sight loss in the UK are female, further research is required to determine how to increase PA participation amongst this population [2].

A final facilitator to being physically active was engaging with a taster day or taster session. A positive early experience with a sport will increase the chances of an individual re-engaging or continuing to engage with PA as they get older [30]. Therefore, the number of taster sessions or taster days offered should increase to promote exposure of sport and PA and to provide individuals with the opportunity to engage in something new.

Whilst this study offers a novel contribution to the research through the inclusion of adults of working age with a VI it is not without its limitations. Despite a desire to include participants of working age recruitment was not capped at 65 and therefore still included perspectives from adults over the age of 65. Further, although it was deemed that data saturation was reached, the findings represent a small sample of individuals specifically from Cambridgeshire who all classed themselves as White British, making the findings geographically and demographically delimited. Although data saturation can be a problematic concept in qualitative research, a good level of conceptual depth was achieved. However, the findings still represent a small sample; therefore, these findings may not be representative of all areas or ethnicities in the UK. Black and ethnic minority groups are at an increased risk of lower levels of PA and an increased risk of eye-disease- and eye-related vision loss, making it essential to understand how to increase PA levels among these demographics [39,40]. An additional limitation is that all participations self-reported as being physically active. Whilst facilitators were discussed it is plausible that someone who is currently inactive might experience different barriers to PA.

## 5. Conclusions

Despite the use of a small and localised sample, this study has reported several barriers that should be considered to increase PA levels for those living with sight loss. Ensuring independence and autonomy for adults with a VI is essential to ensure long-term PA adherence. Localised training must be provided for those who work in the sport and physical activity domain to increase the PA opportunities available and to ensure they are providing positive experiences. However, these changes cannot happen in isolation, and it is essential that public transport and local infrastructure also improves to ensure participants can access their desired activity.

## Figures and Tables

**Table 1:** Descriptive characteristics of participants.

Age	Gender	Ethnicity	Level of Sight Loss	Eye Conditions	Activities
28	Female	White British	Partially sighted	Glaucoma	Goalball
74	Female	White British	Blind	Not specified	Yoga and dance.
32	Female	White British	Partially sighted	Not specified	Judo.
18	Female	White British	Blind	Not specified	Goalball.
44	Male	White British	Blind	Not specified	Horse riding and open-water swimming.
25	Male	White British	Partially sighted	Not specified	Pan-disability football.
33	Male	White British	Blind	Not specified	Goalball.

## Data Availability

The data presented in this study are available on request from the corresponding author.

## Supplementary Materials

The following supporting information can be downloaded at: https://www.mdpi.com/article/10.3390/vision7040070/s1.
