# Supplementary material for: Barriers to and Facilitators of Physical Activity: A Qualitative Study from the Perspective of Individuals Living with Sight Loss in Cambridgeshire
Source: Vision (Basel). 2023 Nov 2;7(4):70. doi: 10.3390/vision7040070 (PMC10661310; doi:10.3390/vision7040070)
Supplement: Supplementary file 1 [file vision-07-00070-s001.zip › vision-2653494-Supplementary Material.pdf]

Focus group protocol

Moderator: Start by introducing myself, and the project:

- Peterborough United in conjunction with ARU are hoping to increase PA opportunities for those living with a VI in and around Peterborough.
- We would like your expert opinion of barriers and facilitators to physical activity as you are the people who we want to attend the sessions.
- Outline how the focus group will run.
- Run over the confidentiality reminder.
- Obtain verbal consent from all participants and remind them that the focus group is going to be audio recorded.

Focus group question guide

Introductory questions:

- All participants will introduce themselves to the group.
- What does being physically active mean to you?
- Why do you/would you participate in physical activity?
    - What is your motivation?

1. Tell us about your experience in accessing your local sporting or physical activity opportunities…

    What makes you attend/ not attend these sessions?

    Please describe the barriers to attending these sessions.

    What is it you like about these sessions.

2. What do you think the challenges to being physically active in your area?

    How do you overcome these barriers?

    How influential is cost in the decision to be physically active?

    How influential is travel in the decision to be physically active?

3. What key things have helped you to get involved in physical activity previously?
4. How do you think sight loss services could support you with being physically active?

    What changes would you like to see made to the current services offered to people with vision impairment?

    e.g., would you like to be brought together more frequently?

5. How do you think we can support you in being physically active?

    What resources would you like to be given to help you increase your physical activity?

6. What mode of physical activity would you like to see offered in Peterborough (e.g., walking, football etc)?

   What would you like offered from these sessions (e.g., transport assistance)

7. What are your goals and expectations of a physical activity session?
8. If time and resources were not object what would your ideal physical activity intervention look like?

   Give participants some time to design their own scheme with probes from the researcher about points they may not address (e.g., cost, transport etc)
